# Proactive use of PROMs in ovarian cancer survivors: a systematic review

**DOI:** 10.1186/s13048-019-0538-9

**Published:** 2019-07-15

**Authors:** Anette Stolberg Kargo, Angela Coulter, Pernille Tine Jensen, Karina Dahl Steffensen

**Affiliations:** 1 Department of Clinical Oncology, Sygehus Lillebalt, Vejle Sygehus, Beriderbakken 4, DK-7100 Vejle, Denmark; 2 Institute of Regional Health Research, University of Southern Denmark, Winsløwparken 19, 3, 5000 Odense, Denmark; 3 Center for Shared Decision Making, Sygehus Lillebalt, Vejle Sygehus, Beriderbakken 4, 7100 Vejle, Denmark; 4 Department of Gynaecology and Obstetrics, Odense University Hospital, J. B. Winsløws Vej 4, 5000 Odense, Denmark; 5 Department of Clinical Research, University of Southern Denmark, Winsløwparken 19, 3, 5000 Odense, Denmark

**Keywords:** Ovarian cancer, Follow-up, Patient reported outcome, Quality of life

## Abstract

**Introduction:**

The use of patient reported outcome measures (PROMs) has increased during the past decade, and the focus on how to use them has resulted in a more proactive application. Studies have shown that proactive use of PROMs during treatment improves patient-clinician communication, leads to better symptom management and may prolong survival among advanced cancer patients. Ovarian cancer is a serious disease in which the majority of patients experience recurrence during the follow-up period and suffer from a number of severe symptoms from underlying disease. This systematic review was conducted to assess the evidence on the proactive use of PROMs as a dialogue tool during follow-up of ovarian cancer patients.

**Results:**

The following databases were searched for relevant literature; PubMed, EMBASE, CINAHL, and the Cochrane Library. The search was conducted in April 2019 without any filters or limits. A total of 643 publications were identified, and 48 studies were found to be potentially eligible. Of the 48 papers, none met the final inclusion criterion of using PROMs proactively as a dialogue tool for ovarian cancer patients during follow-up.

**Conclusion:**

Studies have shown that PROMs can identify otherwise undetected symptoms. Using PROMs proactively during the consultation has been shown to improve symptom management for patients with some other types of cancer. However, we found no studies that had examined the proactive use of PROMs during follow-up of ovarian cancer patients. Future studies should evaluate if the proactive use of PROMs could facilitate a more individualized and more effective follow-up program tailored to the ovarian cancer patient’s needs and preferences.

**Electronic supplementary material:**

The online version of this article (10.1186/s13048-019-0538-9) contains supplementary material, which is available to authorized users.

## Introduction

Worldwide, every year 240,000 women are diagnosed with fallopian tube, primary peritoneal, or ovarian cancer (OC), often in advanced stage with approximately 152,000 dying from the disease. This makes OC the leading cause of gynecological cancer-related deaths. Generally, the initial treatment is extensive surgery and chemotherapy to which most patients respond well. Nevertheless, about 80% of these tumors will recur within a few years after primary treatment and treatment of recurrence is rarely curative [1].

After treatment, most patients enter a five-year follow-up (FU) program, including routine clinical visits, imaging, physical examination, and measurement of the cancer biomarker CA125. The primary purpose of FU is early detection of recurrence, but there is no evidence that routine FU increases survival [2]. It may provide reassurance, but for some routine FU may induce anxiety and fear of recurrence [3]. The literature is sparse on this matter in OC patients which further highlights the need for research on individualized follow up plans based on patient needs and preferences [3, 4].

Patient Reported Outcome Measures (PROMs) are defined as “any report of the status of a patient’s health condition that comes directly from the patient, without interpretation of the patient’s response by a clinician or anyone else”. Patient reported outcomes can be measured by means of standardized and validated questionnaires designed for self-completion by patients or by interview [5]. There are several types of PROMs; generic and disease-specific. Generic PROMs are designed to collect data across disease groups, whereas disease-specific PROMs are designed to collect data on outcomes of specific conditions or diagnoses [6]. Some PROMs combine generic and disease-specific elements to capture a broad assessment of the patient’s health status. PROMs can be used to obtain information on physical, emotional, social, sexual, and cognitive functioning besides evaluating side effects or late effects, global health status, and quality of life (QoL). They are often used in clinical trials to monitor health status and QoL before, during, and after treatments to measure patient-related, subjective outcomes secondary to primary endpoints such as survival.

During the past decade, there has been increased interest in using PROMs in routine practice to monitor patient symptoms during treatment. Their use for clarifying patient needs and monitoring late side effects in long-term survivors has received less attention [7]. Evidence from various cancer diagnoses suggests that the use of PROMs during a clinical visit may improve clinician-patient communication by focusing on issues of greater concern to the patient without prolonging the visit [8]. There is also evidence that clinicians often underestimate late side-effects [9, 10]. Use of PROMs as a dialogue tool, alongside blood samples and imaging, may provide clinicians with more valid and comprehensive knowledge of the patient’s problems [11]. A recent study suggested that active use of PROMs during advanced cancer treatment may even prolong survival [12].

We were specifically interested in the potential use of PROMs to improve follow-up care for ovarian cancer survivors. We therefore undertook a systematic review to determine what is already known about proactive use of PROMs as a dialogue tool during follow-up of these patients.

## Methods

### Data sources and search strategy

We conducted a systematic review to assess the proactive use of PROMs as a dialog or screening tool during follow-up of patients after completion of active treatment (e.g. surgery and chemotherapy) for OC. The review was conducted according to Preferred Reporting Items for Systematic Reviews and Meta-analyses (PRISMA) [13, 14].

During April 2019 a systematic search was conducted by author AK searching the following databases: PubMed, CINAHL, EMBASE, and the Cochrane Library. Relevant articles published between 1974 to April 2019 were identified. Search strategy in PubMed combining Mesh term “Ovarian Neoplasms”, “Patient Reported Outcome Measures”, “Patient Outcome Assessment”, “Health Care Surveys” and key words “Ovarian Cancer”, “ovarian neoplasms”, “patient outcome assessment” and “patient reported outcome”.

The search terms derived after advice from a research librarian and an advisory group including all co-authors who also helped identify additional “grey literature” of relevance to the research question. No filters were applied. The search strategy for databases PubMed, EMBASE, CINAHL and The Cochrane Library is available in Additional file 1. Titles and abstracts of studies retrieved from the search were screened by AK. Reference lists were manually screened to identify additional papers.

### Study selection

Articles were considered eligible if the study participants were OC patients and the proactive use of one or more PROMs during FU was involved. Proactive use of a PROM is defined as data reported by a patient, presented to the clinical staff, and used actively during the consultation as a dialogue tool between patient and clinician.

Studies describing the development of PROMs or PROMs used as a primary or secondary outcome in clinical trials were excluded. Studies were also excluded if PROMs were used to assess the eligibility of patients for chemotherapy, or if they were used to retrospectively identify coping strategies or late side effects with no proactive use.

Review papers were examined for potentially eligible studies that might have been missed in the search strategy. Studies involving multiple cancer sites were excluded if data on OC were not presented separately.

### Identification of relevant articles

The titles and abstracts of all retrieved papers were evaluated to determine the relevance of the study. Full texts were retrieved and examined in case the abstract alone did not provide sufficient information.

### Data extraction

All potentially eligible studies were screened by reviewer AK. Data were extracted on publication details (author, year and country of study, study design, intervention, and sample size) and all PROM-specific data (type of PROM, how and when used) were entered into a pre-designed form.

## Results

**Fig. 1:**
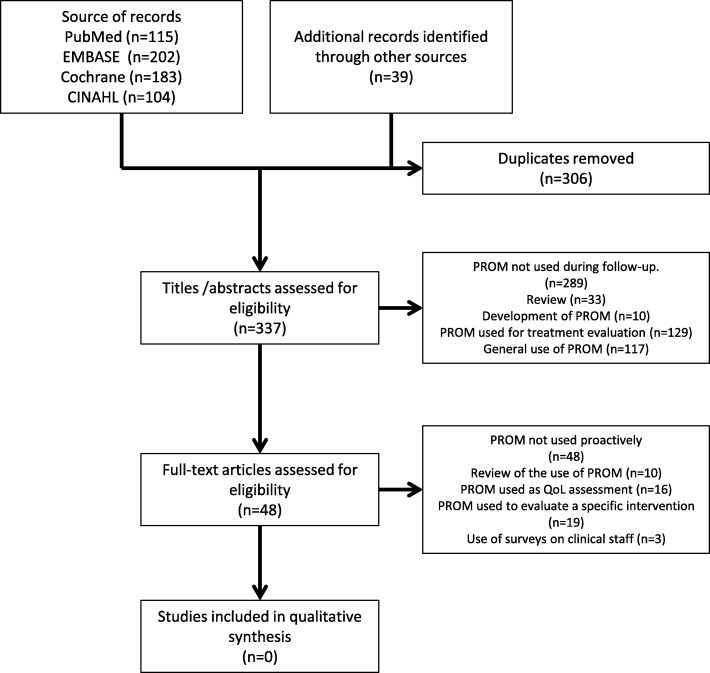
Flow diagram for search and selection process

The search led to the identification of 643 studies, and after removal of duplicates a total of 337 abstracts were selected for detailed examination (Fig. 1). Forty-eight titles/abstracts met the initial selection criteria and full texts of these were obtained for the assessment of eligibility.

Data extraction was performed on the 48 potentially eligible studies, none of which met the final inclusion criterion of using PROMs proactively as a dialogue tool in the follow-up care of OC patients. These papers were excluded for the following reasons: literature review on the use of PROMs (*n* = 10); description of PROM development, papers showing OC data combined with other cancers, and PROMs used to evaluate a specific intervention (*n* = 19); surveys investigating clinical staff’s opinion regarding the use of PROMs (*n* = 3); studies where PROMs were used to assess the patient’s perspective on QoL, or for obtaining prognostic information on life expectancy where the data collected were not used proactively in the care of individual patients (*n* = 16). The search and selection process is shown in Fig. 1. Characteristics of the studies and reason for exclusion are summarized in Table 1.

**Table 1:** Characteristics of studies and reasons for exclusion

Study	Aim and reason for exclusion
Velikova G et al. 2004 [10]	Evaluated the effectiveness of active use of a PROM during treatment and the effect on QoL. This study was performed on mixed cancer sites and data on OC patients were not reported separately. PROMs were primarily used during active treatment.
Hess LM et al. 2012 [15]	Review assessed published literature regarding Health Related QoL among ovarian cancer patients. Reported no studies using PROMs actively during FU.
Hilpert F et al. 2018 [16]	Review examined the current status regard the use of PROM in clinical trials. Highlight that PROM has potential to be used in decision making. Find that PROM most likely will become more important in clinical trials.
Wiering B et al. 2017 [17]	Review assessed the extent to which patients were involved in the development of PROMs and if patients involvement has increased over time. They reported no studies using PROMs actively over time.
Preston N et al. 2015 [18]	Review assessed different PROMs used in gynecological oncology in order to identify the most appropriate PROM. No studies reported using PROMs actively during FU.
Clarke T et al. 2014 [19]	Review compared the benefits of different follow-up strategies in patients with OC. Found one randomized controlled trail regarding follow-up strategies. Highlights the needs for new trails aiming to investigate in different types of FU. Found no study that used PROMs actively.
Kew F et al. 2011 [20]	Review compared the potential benefits of different follow-up strategies in OC survivors. Highlights the needs for trails comparing different FU programs with focus on QoL, survival and cost. Found no studies using PROMs during FU.
Zikos et al. 2016 [21]	Review assessed whether Health Related QoL could provide prognostic information among OC survivors. No studies used PROMs actively during FU.
Ahmed-Lecheheb et al. 2016 [22]	Review examined the literature measuring QoL in patients who survived OC. Find that OC survivors experience a wide range of sequelae that have a negative impact on QoL. Found no studies using PROMs actively.
Detmar SB et al. 2002 [23]	Examined the effect of using PROMs on communications between clinician and patients during active palliative treatment. This study was performed on mixed cancer sites. Study excluded because PROMs were used during treatment and data on OC patients were not reported separately.
King MT et al. 2018 [24]	Aimed to review and validate MOST, a new PROM for OC patients with relapse. PROM used during treatment. MOST can be used to measure symptom benefit and burden during treatment. PROM were not used during FU.
McCorkle R et al. 2011 [25]	Evaluated the effect of support for self-management; the patients were included shortly after surgery and the intervention was applied during active treatment. The intervention was not offered during FU.
Meraner V et al. 2012 [26]	Assessed the course of depressive symptoms, anxiety, fatigue and QoL in patients with OC over the course of chemotherapy and for the 2 first aftercare visits. PROMs were used during treatment and it is unclear if PROMs was used actively and the study was therefore excluded.
Beesley VL et al. 2013 [27]	Aimed to identify risk factors for future unmet needs after first-line treatment for OC. PROMs were used during FU, but the pro-actively use of PROMs is unclear and the study was excluded.
Stewart DE et al. 2001 [28]	Study designed to learn more about self-management of physical health and QoL of OC survivors. Participants had been at least 2 years without treatment. PROMs were used anonymously and not used actively.
Bodurka-Bevers D et al. 2000 [29]	Assessed the prevalence of anxiety and depressive symptoms and QoL problems in OC patients. PROMs were not used proactively.
Greimel E et al. 2011 [30]	Aimed to compare QoL in long-term OC survivors with short-term survivors. PROMs were not used proactively.
Liavaag AH et al. 2007 [31]	Aimed to explore fatigue, QoL, and somatic and mental morbidity between OC with and without relapse. PROMs were not used actively.
Matei D et al. 2009 [32]	Compared late effects of treatment on physical well-being between OGCT survivors and matched controls. PROMs were not used proactively.
Mercieca-Bebber RL et al. 2017 [33]	Investigated if low QoL among OC survivors was associated with earlier study drop out. PROMs were used during FU, but were not used actively.
Guidozzi F. 1993 [34]	Interviewed OC survivors regarding the impact of OC on their QoL. How the answers were used is unclear and the study was removed.
Chase DM et al. 2011 [35]	This paper is an overview of the state of the science of QoL measurement in clinical management. Found no studies using PROMs actively.
Williams LA et al. 2013 [36]	Aimed to develop a new questionnaire for OC patients. Patients were involved in the development of a questionnaire. Their response was not used proactively.
Greimel E et al. 2003 [37]	Aimed to validate the EORTC QLQ-OV28 disease-specific questionnaire. PROMs were not used actively.
Snyder CF et al. 2009 [38]	Focus on implementing PROMs in the clinical setting in general, and not on using PROMs proactively.
Bördlein-Wahl I et al. 2009 [39]	Described the general knowledge of PROMs from a clinician, patient, and scientist point of view. PROMs were not used actively.
Roncolato FT et al. 2017 [40]	Investigated whether baseline QoL score would be prognostic. PROMs were used to assess the effect of an intervention but not used proactively.
Jensen RE et al. 2016 [41]	Accompanies the editorial of E. Basch et al. [42]. PROMs were used to detect change in QoL score and to optimize symptom management during active treatment.
Du Bois A et al. 2005 [43]	Aimed to evaluate if standard care guidelines were followed among OC patients. Surveys were used in clinicians and not OC patients.
Madalinska JB et al. 2007 [44]	Aimed to investigate if baseline characteristic can predict surgery outcome. PROMs were not used proactively.
De Rooij BH et al. 2017 [45]	Aimed to assess the effect of survivorship care plans. PROMs were used during FU but were not used proactively.
Phillips KA et al. 2004 [46]	Used PROMs to investigate if gatekeeper requirements are associated with the utilization of cancer screening, not specific OC patients. PROMs were not used pro-actively.
Beesley VL et al. 2018 [42]	Aimed to identify coping strategies, used by OC survivors. PROM were used to assess their QoL but not used proactively.
Cesario SK et al. 2010 [47]	Aimed to identify OC patients’ worries and fears. PROMs were collected among OC survivors once and not used proactively.
Keim-Malpass J et al. 2017 [48]	PROMs used to identify physical and psychosocial problems at different time points during treatment.
Oberguggenberger A et al. 2016 [49]	PROMs used to identify physical problems after genetic BRCA testing. PROMs were not used actively.
Stukenborg GJ et al. 2016 [50]	Score of PROMs were used to estimate if patients should be given palliative or more aggressive treatment. This study was performed on mixed cancer sites. Data on OC patients were not reported separately.
Rietveld M et al. 2016 [51]	PROMs used to measure the satisfaction with information provided at the time of diagnosis. PROMs were not used pro-actively.
Beesley VL et al. 2011 [52]	PROMs used to identify the amount of physical activity after diagnosis of OC. PROMs were not used proactively.
Greimel E et al. 2019 [53]	PROMs were not used proactively. Describes that PROM can provide important information regard patients QoL during treatment.
Pearman TP et al. 2018 [54]	Evaluated the if the use of a single question “I am bothered by side effects of treatment” in different cancer sites is associated with clinical reported adverse events. PROM were not used proactively.
Astrup GL et al. 2017 [55]	Used PROMs to identify patients at risk of developing symptoms during active treatment, but PROMs were not a part of the FU program. This study was performed on mixed cancer sites.
Basch E et al. 2016 [56]	Evaluated the use of PROMs in different cancer sites as a screening tool, for symptom management during treatment.
Anderson RT et al. 2019 [57]	PROM were not used proactively but used to predict QoL after cancer diagnosis.
Hilarius DL et al. 2008 [58]	This study was performed on mixed cancer sites. Study excluded because PROMs were collected during treatment and data on OC patients were not reported separately.
Shalowitz D. 2015 [59]	Used questionnaires to investigate prognostic issues among clinician.
Kew FM et al. 2006 [60]	Aimed to investigate current practice regarding FU of OC patients. PROMs were not used pro-actively.

We found no studies that proactively used PROMs during follow-up care after ovarian cancer treatment and therefore no qualitative synthesis was applied.

## Discussion

We searched for studies involving the proactive use of PROMs during follow-up after ovarian cancer treatment but found none. Most studies identified were trials evaluating the effect of specific interventions of OC. PROMs have traditionally been used in observational studies and clinical trials to measure the long-term effect of an intervention or to capture toxicity of new therapies. For some other types of cancer the application of PROMs is progressing from being purely a research tool to monitor side effects in clinical trials, to being used proactively in clinical practice for monitoring symptoms during treatment. By incorporating patients’ assessments and priorities in care management it has revealed a higher frequency of unmet needs that otherwise might have been un-recognized [3, 10, 61].However, we found no evidence that this application of PROMs has been tested with OC patients.

de Rooij et al. performed a randomized trial aiming to assess long-term impact of an automatically generated Survivorship Care Plan (SCP) in ovarian cancer patients. The author found that ovarian cancer patients provided with a SCP did not report increased satisfaction with information provision or care [45]. This highlights that optimal follow up plans should be individualized and tailored for each patient and not a automatically pre-defined tool for all patients.

A recent study has shown that pro-active use of PROMs during treatment improves the QoL of cancer patients [56]. Detmar et al. conducted a randomized clinical trial with patients receiving palliative chemotherapy for different cancer types. Incorporating PROM assessments into clinical practice during treatment and actively using them during the consultation improved patient-clinician communication with the potential to increase the awareness of patient needs [23]. The majority of participants were breast cancer patients receiving first line palliative chemotherapy. This population represents a group with a poor prognosis. These findings were supported by those from a randomized clinical trial involving 766 patients with solid tumors assessed by PROMs during active cancer treatment. Routine collection of PROM data was associated with improved survival by a median of 5 months, suggesting that proactive monitoring helps the clinician to intervene before symptoms cause complications [12]. The participants were recruited between 2007 and 2011, and they had different metastatic cancer types (mainly genitourinary cancer), with a poor prognosis. Such a long timeframe for enrollment may have involved a change of treatment, which could have impacted on survival and burden of symptoms. However, the patients completing PROMs received chemotherapy for a longer period than those receiving usual care. This illustrates the potential of PROMs to detect otherwise unrecognized symptoms during treatment in order to prevent serious events at a later stage.

Hansen et al. found that cancer patients experienced a variety of unmet needs during treatment but also during follow-up, and highlights that the patients indicated that they did not received the support that they needed during follow-up. Unmet needs have an important influence on QoL and PROMs used as a screening tool may reveal patients’ perceived unmet need. Interventions to reduce these unmet needs could enhance patient’s quality of life [62]. Ploos van Amstel et al. aimed to explore distress and quality of life in ovarian cancer patients’ during and after treatment, with a mean time since surgery of 3.3 years. The authors found that a third of the participants’ expressed distress. Almost half of the patients with distress indicated that they wanted a referral to a professional [63]. Their findings indicate that ovarian cancer survivors undergo distress and experience symptoms years after they have finished treatment. If PROMs were used proactively during follow-up this could potentially address patients’ needs and lead to higher satisfaction and improved QoL.

Velikova et al. found that if PROM results were shared with physicians before the clinical encounter, discussions of symptoms took place more frequently compared with the control group. A third of the patients were diagnosed with gynecologic cancer, and PROMs were primarily used during active treatment. Only 2 (1%) participants completed PROMs during follow up. It is unclear if they had gynecologic cancer and the findings are not presented separately. This study adds weight to the conclusion that good communication between clinician and patient should be central to the management of cancer patients. Further, the improved communication resulted in better QoL and emotional functioning for some patients [10]. Howell et al. also reported that if the QoL score was shared with the clinician before the consultation, the level of discussion on emotional and psychosocial issues increased [64].

Many studies have investigated the QoL of OC survivors, late side effects, coping strategies, and many other outcomes over time. If PROMs are collected and used actively during treatment, a positive effect on patient-clinician communication, improved QoL, and a better symptom management during treatment is described. The current model for FU of OC patients is characterized by pre-scheduled visits and mainly concerns standard procedures without necessarily taking the patients’ needs and preferences into account. Pre-scheduled visits may take place at a time when the patient is asymptomatic and thereby induce false reassurance. The value of the standard approach to FU is uncertain, and it is not evidence based. Because of the poor prognosis of OC patients in case of relapse, it is essential to optimize the FU program to focus on what matters most to the patient. Furthermore, pro-active use of PROMs will help ensure that patients are met on their own premises and that the time spent during the consultation is used to help the patient with the problems that bother them the most.

Although interest in collecting PROMs in clinical trials and using them actively as a screening or dialogue tool during treatment is growing, our literature search shows that unfortunately, there is not much experience with this for the benefit of ovarian cancer patients. If PROMs are used proactively during consultations, the visit can be tailored to match the individual patient’s preferences and needs. This may be a new approach to routine collection of PROMs to improve patient centered care and individualized treatment.

We are aware of the limitations of this review. Although we used a comprehensive search strategy, it is still possible that some studies may have been missed. Also, data extraction was performed by only one reviewer who made all decisions about inclusion and exclusion. Lastly, it should be noted that any studies published after 14th of April 2019 were not considered in this paper.

## Conclusion

To our knowledge, no studies have used PROMs as a screening or dialogue tool for ovarian cancer survivors during follow-up. The use of PROMs with these patients may help identify otherwise undetected symptoms and improve the management of late side effects. Proactive use of PROMs during follow-up may enhance patient involvement leading to increased satisfaction with care. We believe there is a strong case for further research into this approach to improve the quality of follow-up care of ovarian cancer survivors.

## Additional file

Additional file 1: Seach strategy for databases PubMed, Embase, CINAHL and The Cochrane Library. (DOCX 21 kb)

## Data Availability

All data generated during this study are included in this published article in Table 1 and in supplementary material.

## Abbreviations

FU: Follow-up
OC: Ovarian Cancer
PROMs: Patient Reported Outcome Measures
QoL: Quality of Life
SCP: Survivorship Care Plan
