# Supplementary material for: Proactive use of PROMs in ovarian cancer survivors: a systematic review
Source: J Ovarian Res. 2019 Jul 15;12:63. doi: 10.1186/s13048-019-0538-9 (PMC6631969; doi:10.1186/s13048-019-0538-9)
Supplement: Supplementary file 1 — Seach strategy for databases PubMed, Embase, CINAHL and The Cochrane Library. (DOCX 21 kb) [file 13048_2019_538_MOESM1_ESM.docx]

## Supplementary material 1

**Database PubMed**

1. MH (Ovarian Neoplasms) (79427)
2. TI/AB (ovarian cancer) (48932)
3. TI/AB (ovarian neoplasms) (2707)
4. OR / 1-3 (93167)
5. MH (Patient Reported Outcome Measures) (2768)
6. MH (Patient Outcome Assessment) (7064)
7. MH (Health Care Surveys) (33859)
8. TI/AB (patient outcome assessment) (446)
9. TI/AB (patient reported outcome) (6557)
10. OR / 5-9 (43440)
11. AND 4 + 10 (115)

MH: Mesh heading. TI: Title. AB: Abstract.

**Database EMBASE**

1. MH (Ovary cancer) (75395)
2. TI/AB (ovary cancer) (195)
3. TI/AB (ovarian cancer) (81823)
4. TI/AB (ovary neoplasms) (0)
5. TI/AB (ovarian neoplasms) (1103)
6. OR / 1-5 (128448)
7. MH (Patient Reported Outcome) (42381)
8. MH (Health Care Surveys) (31444)
9. TI/AB (patient reported outcome) (5350)
10. TI/AB (Health Care Survey) (18)
11. OR / 7-10 (73780)
12. AND 6 + 11 (202)

MH: Mesh heading. TI: Title. AB: Abstract.

**Database CINAHL**

1. MH (Ovarian Neoplasms) (88081)
2. TI/AB (ovarian cancer) (58615)
3. TI/AB (ovarian neoplasms) (2639)
4. OR / 1-3 (104607)
5. MH (Patient Reported Outcome) (23326)
6. TI/AB (patient outcome assessment) (49)
7. TI/AB (patient reported outcome) (19379)
8. TI/AB (Health care survey) (1019)
9. OR / 5-8 (24396)
10. AND 4 + 9 (104)

MH: Mesh heading. TI: Title. AB: Abstract.

**Database Cochrane Library**

1. MH (Ovarian Neoplasms) (1731)
2. TI/AB (ovarian cancer) (6457)
3. TI/AB (ovarian neoplasms) (2383)
4. OR / 1-3 (10571)
5. MH (Patient Reported Outcome Measures) (268)
6. MH (Patient Outcome Assessment) (553)
7. MH (Health Care Surveys) (609)
8. TI/AB (patient outcome assessment) (52481)
9. TI/AB (patient reported outcome) (32649)
10. OR / 5-9 (67438)
11. AND 4 + 10 (183)

MH: Mesh heading. TI: Title. AB: Abstract.
